# Peer Relationships and Social Media Use in Adolescents with Body Dysmorphic Disorder

**DOI:** 10.1007/s10802-024-01245-2

**Published:** 2024-09-19

**Authors:** Cassie H. Lavell, Ella L. Oar, Ronald M. Rapee

**Affiliations:** Macquarie University Lifespan Health and Wellbeing Research Centre, Macquarie University, Sydney, Australia

**Keywords:** Body dysmorphic disorder, Anxiety disorder, Victimisation, Peer support, Social media

## Abstract

Body dysmorphic disorder (BDD) is a common and debilitating disorder in adolescents, yet there is little research on the disorder in young people. The current study aimed to investigate peer relationship factors in 26 adolescents (aged 12 to 17 years) with BDD, compared to 27 adolescents with anxiety disorders and 25 adolescents without mental disorders. Participants completed self-report measures on peer appearance and general victimisation, peer support, appearance co-rumination and social media use. Adolescents with BDD and anxiety disorders perceived significantly less peer support than adolescents in the non-clinical control group. Although the frequency of perceived appearance and general victimisation did not differ significantly between groups, adolescents with BDD reported significantly more distress due to appearance victimisation than the non-clinical control group. Adolescents with BDD and anxiety disorders reported spending more time on social media than the non-clinical control group, and the BDD group engaged in significantly more online appearance comparisons than both the anxiety and control group. The relationships between BDD, victimisation, social media use, and other peer factors require further empirical investigation.

## Introduction

BDD is a common and debilitating mental health disorder characterised by a preoccupation with perceived flaws in physical appearance (American Psychiatric Association, 2022). It is estimated to affect approximately 2% of adults, and is associated with severe impairments in functioning (Phillips et al., 2005) including social functioning (Phillips, 2000). BDD symptoms commonly first emerge in adolescence (Gunstad & Phillips, 2003; Phillips et al., 1993). Prevalence rates for BDD in adolescents are comparable to those in adult populations (Mayville et al., 1999; Möllmann et al., 2017; Rief et al., 2006), with up to 1.7% of Australian adolescents meeting criteria for BDD (Schneider et al., 2017). While the clinical features of BDD in youth are similar to adults, research suggests that symptoms may be more severe in young people, they may have poorer insight, more delusional beliefs, and higher rates of suicide attempts than their adult counterparts (Albertini & Phillips, 1999; Phillips et al., 2006). Adolescents with BDD are at significant risk for suicidality, with 67–81% of adolescents with BDD reporting lifetime suicidal ideation, and 21–44% attempting suicide (Albertini & Phillips, 1999; Phillips et al., 2006).

Despite the severity and impact of BDD in adolescents, there is a paucity of research into the aetiology of the condition in youth. Cognitive-behavioural models of BDD (Wilhelm et al., 2012; Neziroglu et al., 2008; Veale & Neziroglu, 2010) propose a range of possible aetiological factors, including genetic vulnerability (Enander et al., 2018; Monzani et al., 2014) and differences in cognitive and emotional processing (Johnson et al., 2018). These models also highlight that aversive or traumatic childhood experiences may be developmental risk factors for BDD (Buhlmann et al., 2011; Longobardi et al., 2022; Malcolm et al., 2021b). For example, research suggests that a history of school-based bullying is common among adults with BDD (Weingarden et al., 2017), which indicates that peer experiences could serve as risk factors for the development of BDD during adolescence. Once symptoms emerge, BDD is thought to be maintained via processes such as the overvaluation of physical appearance, making upward appearance comparisons, and avoidance and safety behaviours. Hence, adolescents’ social context, such as the peer appearance culture and appearance comparisons on social media, could also maintain symptoms of BDD during this developmental period.

Indeed, over the course of adolescence peer relationships become increasingly salient, and research has shown that adolescents’ peer relationships influence their body image, including the development of body dissatisfaction (Webb & Zimmer-Gembeck, 2014; Fardouly & Vartanian, 2016). However, the role of peer relationships in adolescent BDD has been under researched to date. Drawing from cognitive-behavioural models of BDD and the broader body image literature, the current study aimed to examine peer-related experiences among adolescents with BDD including peer victimisation, social support, co-rumination, and social media use. Understanding environmental correlates of BDD in adolescents will provide direction for future research regarding the development and maintenance of BDD in young people. This research is critical given that many young people with BDD remain symptomatic after receiving current evidence-based treatments (Krebs et al., 2017).

### Victimisation

Negative peer interactions such as rejection and victimisation are known correlates of adolescent psychopathology, including depression and anxiety (Epkins & Heckler, 2011; Rapee et al., 2019). Consistent with cognitive-behavioural models of BDD, there is evidence to suggest aversive social experiences in childhood may be relevant to the development and maintenance of BDD in adolescents (Longobardi et al., 2022). Adults with BDD retrospectively report more instances of teasing during their childhood compared to adults without BDD (Buhlmann et al., 2007), and also recall these experiences more vividly and as being more traumatic (Buhlmann et al., 2011). Furthermore, in a study of patient-identified events associated with the development of BDD, the most common type of event identified in a sample of 165 adults with BDD was bullying (Weingarden et al., 2017). The authors also found that adults who specifically attributed bullying experiences to the development of their BDD had poorer psychosocial outcomes than those who reported another type of triggering event (Weingarden et al., 2017). While these studies indicate retrospective accounts of childhood bullying are common among adults with BDD, fewer studies have explored bullying experiences among adolescents exhibiting features of BDD.

In non-clinical samples of adolescents, appearance-based victimisation and general victimisation by peers have consistently been found to be associated with heightened body dysmorphic symptoms (Fabris et al., 2021; Lavell et al., 2018; Webb et al., 2015, 2016; Zimmer-Gembeck et al., 2018). To date, there are no published studies examining peer relationships among adolescents with a clinical diagnosis of BDD, however in a study of children 7 to 10 years, Neziroglu and colleagues (2018) found that children with a likely diagnosis of BDD (based on a self-report measure of DSM-5 criteria for BDD; *n* = 23) were more likely to report being bullied than those without a mental disorder (*n* = 158). However, children with BDD reported significantly less bullying than a comparison sample of children with OCD (*n* = 10) and had comparable rates of bullying as a clinical control group (i.e., children with anxiety, depression, and externalising disorders; *n* = 28). Interestingly, in this study, children with BDD were also more likely to report being perpetrators of bullying than children with OCD and children without psychiatric diagnoses (Neziroglu et al., 2018). This study was limited by the clinical group’s small sample size. Further, the instrument used was a measure of general bullying and specific appearance-related victimisation was not assessed. In summary, research suggests a possible relationship between adolescent BDD and peer victimisation experiences, however studies comparing adolescents with a clinical diagnosis of BDD to control groups are very much needed.

### Peer Support

Just as peer victimisation is a risk factor for psychopathology in adolescents, conversely, social support from peers can serve a protective role. Peer support is associated with lower levels of anxiety and depression in youth (Rueger et al., 2016; Van Droogenbroeck et al., 2018). Further, peer support may buffer the effects of adversity on psychopathology and suicidal ideation (Eze et al., 2021; Fredrick et al., 2018; Mackin et al., 2017; Rueger et al., 2016).

While it has been established that people with BDD experience impairments in social functioning (Phillips, 2000; Phillips et al., 2005a), there is limited research on their perceptions of social support. In a study of 400 adult participants who self-reported symptoms consistent with a diagnosis of BDD, more perceived social support from friends was associated with less severe BDD symptoms in both men and women (Marques et al., 2011). In non-clinical studies of adolescents, lower perceptions of peer acceptance (i.e., how well-liked they are by peers) are reported by youth with higher BDD symptoms (Mastro et al., 2016; Webb et al., 2016). However, a potential protective effect of peer support is yet to be examined in a clinical sample of adolescents with BDD.

### Appearance Co-Rumination

While social support from peers is a predictor of emotional well-being, certain types of peer support and interactions may not be beneficial. Co-rumination refers to the dyadic process of engaging in repetitive discussions regarding problems, including encouraging problem-talk and dwelling on negative experiences (Rose, 2002, 2021). While the process is reported to include positive aspects (e.g., increased social support; Rose, 2002) it is primarily considered unproductive, and has been shown to be associated with internalising problems including anxiety and depression (Spendelow et al., 2017). There is growing evidence that body-related co-rumination, negative body talk, and appearance conversations within relationships are associated with body dissatisfaction among adults and adolescents (Mills & Fuller-Tyszkiewicz, 2017; Rudiger & Winstead, 2013; Webb & Zimmer-Gembeck, 2014). Research in non-clinical adolescent samples suggests peer appearance talk is also associated with BDD symptomatology. For example, in a study of 387 adolescents, Mastro et al. (2016) found participants who scored high on a measure of BDD symptoms reported more frequent appearance-related conversations with their friends than other adolescents. To date, there are no studies comparing appearance co-rumination in adolescents with BDD versus those without BDD.

### Social Media

Almost all adolescents in Western countries utilise some form of social media, and most report feeling as though their usage keeps them connected to their friends (Office of the eSafety Commissioner, 2018; Pew Research Center, 2022). However, some studies suggest there may be a relationship between social media use and adolescent well-being, whereby greater time spent on social media has been found to be associated with body dissatisfaction (Fardouly & Vartanian, 2016; Holland & Tiggemann, 2016; Ryding & Kuss, 2020; Saiphoo & Vahedi, 2019; Vandenbosch et al., 2021) and other mental health concerns such as anxiety and depression (Fassi et al., 2023; Keles et al., 2020; Piteo & Ward, 2020; Twenge et al., 2018). Much of this research however is limited by a reliance on cross-sectional data, while longitudinal studies have failed to establish a causal relationship between increased time spent on social media and worse mental health outcomes over time (Coyne et al., 2020; Ferguson et al., 2022; Heffer, Good, Daly, MacDonell, & Willoughby, 2019; Jensen et al., 2019). In light of this, a growing body of research has examined whether specific social media activities may be more important than time spent on social media in predicting adolescent well-being. For example, research suggests that making upward appearance comparisons, viewing idealised images, and taking and editing “selfies” may be associated with more negative body image (Fardouly et al., 2015; Fardouly & Vartanian, 2015; Tiggemann et al., 2020). There is also evidence from a meta-analysis of longitudinal studies that exposure to idealised images online may have a negative causal impact on body image (de Valle et al., 2021). Conversely, emerging experimental research in young women indicates that certain activities on social media, such as viewing “body positive” posts and natural or “no makeup” images, may be protective against body dissatisfaction (Cohen et al., 2019; Dhadly et al., 2023; Fardouly & Rapee, 2019; Tiggemann & Anderberg, 2020).

Although there is limited research on social media use and BDD specifically, in their systematic review, Ryding and Kuss (2020) proposed that given its relationship with body dissatisfaction, frequent social media use may potentially be related to BDD symptomology. Indeed, in a case study of a 21-year-old woman with BDD, Khanna and Sharma (2017) highlighted how social media usage can become excessive for patients dealing with BDD, including frequent checking of social media and analysing and re-taking of “selfies” to manage distress. With the limited research to date, it is unclear how prevalent excessive social media use and related appearance comparisons are among young people with BDD. Research examining the social media activities of adolescents with BDD may broaden our understanding of environmental correlates of the disorder.

In sum, existing research suggests associations between body dysmorphic symptoms and a range of peer-related experiences. However, a lack of inclusion of clinical samples with BDD and a lack of clinical comparison groups limits current conclusions. Hence, there is a need for research into peer relationships and social media use in clinical samples of adolescents with BDD to inform the development of theoretical models. Hence, the current study aimed to investigate the social context of adolescents with BDD compared to two control groups: (1) adolescents with anxiety disorders and (2) adolescents without mental disorders. Anxiety disorders were selected as an internalising control group, given the diagnostic overlap between BDD and a number of anxiety disorders, including social anxiety disorder (Fang & Hofmann, 2010). Moreover, the prevalence of anxiety disorders makes this a feasible comparison group to recruit.

It was hypothesised that, (1) compared to the anxious and non-clinical groups, adolescents with BDD would report more frequent appearance victimisation from peers, more distress regarding appearance victimisation, higher bullying perpetration, and more appearance co-rumination with their peers. Regarding social media use, it was hypothesised that (2) adolescents with BDD, compared to anxious and non-clinical adolescents, would report engaging in more appearance comparisons and “appearance investment” (e.g., selfie taking, editing, and removing photos) when using social media and viewing less body positive content on social media. It was also predicted that (3) adolescents with BDD would report spending more time on social media, having less social support from peers, and experiencing more general victimisation from peers compared to non-clinical adolescents, but that these factors would be comparable to the levels reported by anxious adolescents.

## Method

### Participants

Participants were 78 adolescents aged between 12 and 17 years (*M*_*age*_ = 14.05, *SD* = 1.57). The participants comprised three groups: (1) a BDD group (*n* = 26), (2) a control group of adolescents with anxiety disorders (*n* = 27), and (3) a non-clinical control group (*n* = 25). Participants were recruited via advertising through health professionals, school newsletters, and social media. Families self-referred to the study.

Participants in the BDD group (*M*_*age*_ = 14.54, *SD* = 1.61) met criteria for a primary diagnosis of BDD based on the Diagnostic and Statistical Manual for Mental Disorders, 5th Edition Text Revision (DSM-5-TR; American Psychiatric Association, 2022). The clinical control group included adolescents (*M*_*age*_ = 13.78, *SD* = 1.67) with a primary DSM-5-TR anxiety disorder diagnosis. Primary diagnoses of participants in clinical control group included social phobia (40.7%), specific phobia (25.9%), generalised anxiety disorder (GAD; 9.6%), and separation anxiety disorder (SAD; 3.7%). Across both the BDD and anxiety groups comorbidity with other mental disorders was permissible provided they were secondary diagnoses with one exception: adolescents in the anxiety disorder group were excluded if they had comorbid BDD. Due to the high comorbidity between BDD and anxiety disorders (up to 66%; Phillips et al., 2006) it was not possible to obtain a BDD-only sample. The non-clinical control group included adolescents (*M*_*age*_ = 13.84, *SD* = 1.34) who did not meet criteria for any DSM-5-TR psychiatric diagnosis.

The majority of the overall sample were girls (70.5%) and cisgender (96.2%). Three participants (one participant in each group) were transgender or non-binary. Of the overall sample, 85.9% were White, 6.4% were Asian, 2.6% were Middle Eastern, and 1.3% were Aboriginal.

### Measures

#### Diagnostic Status

Participant DSM-5-TR diagnostic status was determined using the Mini International Neuropsychiatric Interview for Children and Adolescents – Child Version (MINI-Kid; Sheehan et al., 2010) and the revised adolescent version of the BDD Diagnostic Module (Phillips, 2005, 2017). Both diagnostic interviews were administered with the adolescent via Zoom by a postgraduate-trained psychologist. Primary diagnosis was determined by ranking with the young person which diagnosis caused them the most distress and/or interference. Research indicates that diagnostic interviews conducted via videoconference or telephone are comparably reliable and valid to face-to-face interviews (Elford et al., 2000; Lyneham & Rapee, 2005).

The MINI-Kid is a structured diagnostic interview to assess the presence of a range of DSM-5 mental disorders in children and adolescents 6 to 17 years, including mood disorders, anxiety disorders, OCD, tic disorders, eating disorders, post-traumatic stress disorder, externalising disorders, attention-deficit/hyperactivity disorder (ADHD) and the presence of autistic traits. The interview has demonstrated substantial interrater and test-retest reliability, as well as high sensitivity and specificity (Sheehan et al., 2010). Given the lack of coverage of BDD in the MINI-Kid, the BDD Diagnostic Module (Phillips, 2005) was added, which is a widely used semi-structured diagnostic interview that maps onto the DSM-5-TR criteria for BDD. The adult version for DSM-IV demonstrated excellent interrater reliability (Phillips, 2005). In the current study, 15% of BDD Diagnostic Module interviews were reviewed for interrater reliability, and there was 100% agreement between raters.

#### Appearance Victimisation

The appearance-related teasing subscale of the Perceptions of Teasing Scale (POTS; Thompson et al., 1995) was used to measure adolescents’ perceptions of appearance-based victimisation by peers. The POTS appearance teasing items were originally developed to assess weight-based teasing, therefore items were adapted to reflect general appearance concerns (e.g., “*People made fun of you because you were heavy*” was changed to “*People made fun of you because of your looks*”). This adapted version of the POTS has been used previously in adolescent samples (e.g., Webb et al., 2015) and a study of adults with BDD (Buhlmann et al., 2007).The 6-item scale measures both the frequency of perceived appearance-based victimisation (total score range = 6 to 30) and the associated distress (range = 0 to 30). Reliability in the current sample was *α* = 0.88 for the frequency subscale and *α* = 0.88 for the distress subscale, which is comparable to the reliability of the original weight-based teasing scale (Thompson et al., 1995).

#### General Victimisation

The Revised Olweus Bully/Victim Questionnaire (OBVQ; Olweus, 1996) was used to measure adolescents’ perceptions of general peer victimisation as well as their own bullying perpetration. Peer victimisation was measured using 6-items (total score range = 6 to 30), and bullying perpetration was measured with a single item “*How often have you taken part in bullying another student(s) at school in the past couple of months?”* (range = 1 to 5). In the current sample, reliability for the OBVQ (victimisation) was *α* = 0.85.

#### Peer Support

Participants were asked to report the number of close friends they had on a 6-point scale (0 = *None* to 5 = *10 or more*). The 12-item close friend subscale of the Child and Adolescent Social Support Scale (CASSS; Malecki & Demaray, 2002) was used to measure adolescents’ perceived social support from peers. Participants rated how often they received various types of social support from their close friend (1 = *Never* to 6 = *Always;* total score range = 12 to 72), as well as how important the type of support was to them (1 = *Not important* to 3 = *Very important;* range = 12 to 36). Cronbach’s alpha for the CASSS in the current study was *α* = 0.95 for the frequency subscale and *α* = 0.91 for the importance subscale.

#### Appearance Co-Rumination

A modified version of the Co-Rumination Questionnaire (Rose, 2002) was used to measure participants’ appearance-based co-rumination with peers, based on the 12-item appearance-focused adaptation of the scale by Rudiger (2010). While Rudiger (2010) asked participants to rate items in relation to “*problems related to physical appearance or dieting/exercise*”, in the current study participants were instructed to rate items based on *“problems related to looks i.e.*,* unhappiness or dissatisfaction with one’s body (e.g.*,* weight/shape*,* hair*,* face*,* legs*,* arms*,* etc.).”* The scale included 12-items rated from 1 (*Not at all true*) to 5 (*Really true*), with total scores ranging from 12 to 60. Cronbach’s alpha in the current study was *α* = .93, which is comparable to the reliability of previous versions of the scale (Rose, 2002; Rudiger, 2010).

#### Social Media Use

Participants completed a self-report Social Media Questionnaire (SMQ) developed by Fardouly and colleagues (2020). They were asked to report the perceived duration of the time they spend browsing social media on a typical weekday (ranging from 0 = *No time* to 10 = *10 h or more*). They also rated how often (0 = *Never* to 5 = *Very often [daily]*) they engaged in various behaviours while using social media, including posting images or videos (2 items; range = 0 to 10), making appearance comparisons (3 items; range = 0 to 15), and behaviours related to “appearance investment” including taking selfies, editing selfies, re-taking selfies, and removing images they didn’t like or that did not get enough comments or likes (5 items; range = 0 to 25). A single item also measured the frequency participants viewed “body positive” images on social media (range = 0 to 5). Reliability for the posting subscale was low at *α* = 0.55, but high for the appearance comparison (*α* = 0.95) and appearance investment (*α* = 88) subscales.

### Procedures

The study was conducted as part of a broader study of factors associated with BDD in adolescents. The Macquarie University Human Research Ethics Committee approved the study (approval no: 52023902649677). Participants were initially screened via telephone call with the parent. Participants and their parents/caregivers gave informed consent to participate in the study before attending the diagnostic interview via a Zoom meeting. Following completion of the diagnostic interviews, adolescents who were deemed eligible for the study completed online self-report questionnaires, while parents completed questions regarding demographic characteristics (including household income and participant race/ethnicity). Families were reimbursed with a $30 gift voucher for their participation in the study.

### Statistical Analyses

Analysis of variance (ANOVA) and chi-square tests were conducted using SPSS version 28 to examine group differences in demographic characteristics. Hypotheses 1 to 3 were tested using one-way ANOVAs to examine group differences in victimisation, social support, appearance co-rumination and social media use. Significant main effects were followed with Bonferroni post-hoc comparisons. Between-groups effect sizes (Cohen’s *d*) were calculated with the Campbell Collaboration effect size calculator (https://campbellcollaboration.org/), inputting sample sizes, means, and SDs. To correct for multiple comparisons, the Benjamini–Hochberg procedure was used (Benjamini & Hochberg, 1995) with a paper-wide false discovery rate of 0.05, which resulted in an adjusted *p* = .009.

## Results

One participant in the BDD group did not complete the appearance co-rumination or social media questionnaires, therefore the final BDD sample for analyses conducted using these scales was *n* = 25. There was no other missing data. The prevalence of co-occurring DSM-5-TR diagnoses (excluding primary diagnoses) in the BDD and anxiety groups are presented in Table 1. There were no significant differences in demographic characteristics between participant groups (see Table 1).

As shown in Table 2, no significant group main effects were found for adolescents’ perceptions of the frequency of appearance victimisation, general victimisation, appearance co-rumination, importance of social support, or bullying perpetration. However, significant main effects were observed for participants’ distress due to appearance victimisation and perceived social support from close peers.

**Table 1:** Participant characteristics among BDD, clinical control and non-clinical control groups

	BDD (*n* = 26)	Clinical control (*n* = 27)	Non-clinical control (*n* = 25)	Statistic	*p*
Age *M (SD)*	14.54 (1.61)	13.78 (1.67)	13.84 (1.34)	*F*(2,75) = 1.93	0.152
Gender *n (%)* Boys	6 (23%)	7 (26%)	8 (32%)	*χ*^*2*^ (2, *N* = 78) = 0.54	0.765
Race/Ethnicity *n (%)* White	22 (88%)	26 (96%)	20 (80%)	*χ*^*2*^ (2, *N* = 77) = 3.34	0.188
Household Income *n (%)* above $80,000 AUD	21 (84%)	24 (89%)	24 (96%)	*χ*^*2*^ (2, *N* = 77) = 1.96	0.376
Comorbidity *n (%)*					
MDD	11 (42%)	1 (4%)			
Social Phobia	16 (62%)	6 (22%)			
GAD	10 (38%)	3 (11%)			
Specific Phobia	8 (31%)	9 (33%)			
Panic Disorder	3 (12%)	3 (11%)			
SAD	1 (4%)	1 (4%)			
PTSD	4 (15%)	0 (0%)			
OCD	4 (15%)	6 (22%)			
ADHD	10 (38%)	11 (41%)			
ODD	2 (8%)	2 (7%)			
Tic Disorder	2 (8%)	1 (4%)			
Anorexia Nervosa	1 (4%)	0 (0%)			
BED	1 (4%)	0 (0%)			
Autism traits	25 (96%)	24 (89%)			

Note AUD = Australian Dollars; MDD = major depressive disorder; GAD = generalised anxiety disorder; SAD = separation anxiety disorder; PTSD = post-traumatic stress disorder; OCD = obsessive-compulsive disorder; ADHD = attention-deficit/hyperactivity disorder; ODD = oppositional defiance disorder; BED = binge eating disorder

Post-hoc analyses demonstrated the BDD group reported significantly more distress from appearance victimisation (*p* = .001, *d* = 0.97, 95% CI [0.39,1.55]) compared to the non-clinical control group. The difference between the BDD and the clinical control group on distress from appearance victimisation (*p* = .093, *d* = 0.53, 95% CI [-0.01,1.08]) was non-significant. Both the BDD group (*p* = .005, *d* = -0.96, 95% CI [-1.54,-0.38]) and the clinical control group (*p* =.007, *d* = -0.92, 95% CI [-1.49,-0.35]) reported receiving less social support from close friends than the non-clinical control group. The BDD group and the clinical control group did not differ significantly on perceived social support (*p* = 1.00, *d* = -0.03, 95% CI [-0.57,0.51]).

**Table 2:** Group differences on victimisation, social support, co-rumination and social media use

	BDD	Clinical Control	Non-Clinical Control			
	*M (SD)*	*M (SD)*	*M (SD)*	Statistic	*p*	*η*_*p*_^*2*^
	*n* = 26	*n* = 27	*n* = 25			
POTS Frequency	12.38 (6.05)	10.41 (4.09)	8.64 (3.45)	*F*(2, 75) = 4.11	0.020	0.10
POTS Distress	11.69^a^ (10.09)	7.19^ab^ (6.43)	4.00^b^ (4.78)	*F*(2, 75) = 6.87	**0.002**	**0.16**
OBVQ Victim	9.96 (4.97)	8.67 (4.38)	7.00 (1.35)	*F*(2, 75) = 3.63	0.031	0.09
OBVQ Bully	1.04 (0.20)	1.15 (0.36)	1.08 (0.40)	*F*(2, 75) = 0.74	0.479	0.02
No. of Close Friends	2.31 (1.29)	2.41 (1.01)	3.00 (0.96)	*F*(2, 75) = 2.17	0.058	0.07
CASSS Frequency	48.31^a^ (13.27)	48.74^a^ (13.32)	59.56^b^ (9.84)	*F*(2, 75) = 6.84	**0.002**	**0.15**
CASSS Importance	26.42 (6.12)	24.81 (5.42)	26.40 (5.99)	*F*(2, 75) = 0.66	0.521	0.02
	*n* = 25	*n* = 27	*n* = 25			
ACRS	21.12 (10.01)	20.48 (7.77)	22.32 (11.92)	*F*(2, 74) = 0.23	0.799	01
SMQ Time	5.84^a^ (2.10)	5.78^a^ (1.78)	4.08^b^ (2.10)	*F*(2, 74) = 6.35	**0.003**	**0.15**
SMQ Posting	2.88 (1.45)	3.37 (1.36)	2.88 (1.09)	*F*(2, 74) = 1.22	0.300	0.03
SMQ Comparisons	11.24^a^ (4.25)	6.29^b^ (3.35)	4.52^b^ (2.60)	*F*(2, 74) = 25.34	**< 0.001**	**0.41**
SMQ Investment	8.24 (5.60)	8.04 (3.85)	5.52 (1.23)	*F*(2, 74) = 3.80	0.027	0.09
SMQ Body Positive	2.48 (1.26)	2.26 (1.23)	1.56 (0.92)	*F*(2, 74) = 4.39	0.016	0.11

Note Means sharing superscripts are not significantly different at *p* <.009; POTS = Perceptions of Teasing Scale; OBVQ = Olweus Bully/Victim Questionnaire; CASSS = Child and Adolescent Social Support Scale; ACRS = Appearance Co-Rumination Scale; SMQ = Social Media Questionnaire

There were also significant main effects of group on time spent on social media use and online appearance comparisons, although main effects for appearance investment, frequency of posting on social media, and viewing “body positive” content were non-significant. Post-hoc analyses showed that adolescents within the BDD (*p* = .008, *d* = 0.84, 95% CI [0.26,1.42]) and clinical control groups (*p* = .009, *d* = 0.88, 95% CI [0.31,1.44]) reported spending significantly more time on social media than the non-clinical control group, whereas the BDD group and clinical control group did not differ significantly (*p* = 1.00, *d* = -0.03, 95% CI [-0.51,0.58]). Adolescents with BDD reported making significantly more appearance comparisons on social media compared to the clinical (*p* <.001, *d* = 1.30, 95% CI [0.70,1.90]) and non-clinical control groups (*p* <.001, *d* = 1.91, 95% CI [1.24,2.58]), while the two control groups did not differ significantly (*p* = .206, *d* = 0.59, 95% CI [0.03,1.14]).

## Discussion

The current study compared adolescents aged 12 to 17 years with BDD against clinical and non-clinical control groups on peer-related experiences. To the authors’ knowledge, this is the first study to examine peer relationships among a sample of adolescents with a clinical diagnosis of BDD. Contrary to the study hypotheses, differences in the frequency of perceived appearance-based and general victimisation between groups were non-significant. Moreover, adolescents with BDD were no more likely to be perpetrators of bullying than the non-clinical control group or the anxious group. These findings are inconsistent with previous research in adults with BDD, who retrospectively report more bullying experiences in childhood than the general population (Buhlmann et al., 2007, 2011). Results are also inconsistent with a study of bullying in a sample of children with BDD symptoms (Neziroglu et al., 2018), in which children with BDD symptoms reported more victimisation than non-clinical controls and more bullying perpetration than clinical and non-clinical controls. Differences across studies may reflect differences in perceived victimisation between age groups, whereby the prevalence of bullying tends to peak in early adolescence (Hymel & Swearer, 2015). The mean age of participants was 14.05 years in the current study, and 7.95 years in Neziroglu et al. (2018). Perhaps an early adolescent peak in victimisation means group differences were harder to detect in the current sample, as the non-clinical group may have also been experiencing an increase in victimisation. Importantly, adolescents with BDD reported experiencing significantly more distress due to appearance victimisation than non-clinical controls, while anxious adolescents fell between the BDD and non-clinical groups in terms of distress. This suggests that although the prevalence of victimisation may be similar across groups, adolescents with BDD may experience heightened distress from appearance-based victimisation. This is consistent with Buhlmann et al. (2011), who found that adults with BDD remembered teasing experiences more vividly and as more traumatic than adults without BDD. Adults with BDD may also be more likely to remember incidents of bullying in childhood relative to adults without BDD due to the distress they experienced.

From the reverse perspective, adolescents with BDD and anxiety disorders reported similar levels of social support from close peers, but this was significantly less than the non-clinical control group. This suggests that perceptions of low social support may be associated with internalising psychopathology in adolescents, including BDD. Adolescents across all groups considered social support from close friends equally important, and reported having the same number of close friendships, indicating that adolescents with BDD and anxiety disorders may value peer support equally and have the same quantity of friendships, but do not perceive the same quality of peer support, as other adolescents. Given social support is considered to be protective from internalising symptoms for youth experiencing victimisation (Davidson & Demaray, 2007), a perceived absence of peer support may perpetuate distress in BDD, due to fewer opportunities to receive emotional support for bullying experiences, to be helped with problem-solving, and to feel understood and well-liked by peers. Adolescents who perceive they have less social support from their peers, and who are more distressed by perceived bullying experiences, may experience feelings of rejection and defectiveness as a result. This may in turn drive them to make attempts to avoid further rejection and “fit in” socially by camouflaging or attempting to change parts of themselves, including their appearance (e.g., with clothing, makeup and cosmetic procedures). Perceptions of poor peer support and distress from victimisation may also contribute to avoidance behaviours frequently performed by adolescents with BDD, such as avoiding school, parties, and other social events (Rautio et al., 2022), as these situations may not feel safe or supportive to them. As a consequence of safety behaviours and avoidance, adolescents with BDD may have even fewer opportunities to test their beliefs and overcome their anxiety.

Contrary to hypotheses, no differences in adolescents’ perceptions of appearance co-rumination among peers were observed. This finding is inconsistent with Mastro et al. (2016), who found that adolescents “at risk” of BDD based on a self-report measure reported more frequent appearance conversations with their friends compared to other adolescents. In the current study, the appearance co-rumination measure reflected peer conversations about perceived “problems” with appearance, while the scale used by Mastro et al. (2016) included items about general appearance-related conversations (Jones et al., 2004). Therefore, it is possible that adolescents with BDD may engage in appearance “talk” with friends, but not necessarily negative co-rumination about their own appearance. Indeed, BDD has been described as a “secret” illness, as sufferers often do not disclose their appearance concerns to others (Grant et al., 2001; Phillips, 2005). Hence, to further explore whether a peer appearance culture is associated with BDD in young people, future research should examine various types of appearance-related talk amongst peers in a clinical sample, such as peer conversations around the importance of appearance, appearance ideals (e.g., celebrities), the appearance of others (e.g., criticism and/or comparisons), and the use of camouflaging behaviours (e.g., clothing and makeup).

This study also highlights the potential role of certain social media activities in the clinical presentation of BDD in adolescents. Adolescents with BDD and anxiety disorders in the current study reported spending more time on social media than adolescents without mental disorders. As hypothesised, the BDD group reported engaging in more frequent online appearance comparisons than the anxious and non-clinical groups. Findings suggest that more time spent on social media may be a transdiagnostic process in adolescents with internalising psychopathology, while more frequent online appearance comparisons may be an especially important process for adolescents with BDD. Although further research is needed, it is possible that online appearance comparisons serve to maintain BDD symptoms. Moreover, if adolescents with BDD are spending more time on social media than their typically developing peers, they may consume more idealised images, possibly triggering more frequent appearance comparisons. Indeed, offline appearance comparisons are considered an important maintenance factor in BDD, whereby the individual compares specific features of their appearance to others’, determines that they are less attractive, leading to increased focus on appearance and heightened distress (Veale & Neziroglu, 2010; Neziroglu et al., 2008). Hence, by viewing appearance-related content and making appearance comparisons online, adolescents with BDD may inadvertently reinforce their own preoccupation and distress in a similar way to when making offline comparisons. Given the study was cross-sectional, future prospective studies are needed to test these causal hypotheses.

Contrary to hypotheses, there were no significant differences in posting on social media, social media investment, or viewing body positive content online. This suggests adolescents with BDD in the current study were no more likely than other adolescents to post images online to social media, or to edit, re-take or remove images they were unhappy with from social media. Moreover, viewing body positive content did not emerge as a possible protective factor in the current study. As much of the research to-date exploring online appearance investment and body positive content has involved non-clinical samples, results of the current study suggest these factors may not be as important in differentiating adolescents with clinically significant body image concerns such as BDD from other adolescents. Group means for the frequency of viewing body positive were generally low in all groups, with a non-significant trend toward the BDD group viewing this content somewhat more frequently. Although there has been a recent popularisation of body positive content on social media (Cohen et al., 2019, 2021), to the authors’ knowledge the rates at which adolescents view this content relative to adults is unknown. Moreover, there may be a difference between the effect of passive viewing of body positive content versus active searching for content. In a community sample of 1,530 Czech adolescents ages 13 to 18, Kvardova et al. (2022) found a significant moderating role of the frequency of intentional searching for body positive content online, whereby intentional searching strengthened the relationship between viewing this content and body satisfaction. The current study did not differentiate between intentional searching and passive viewing of body positive content, which may be an area for future research on adolescents with BDD.

### Clinical Implications

Current cognitive-behavioural therapy (CBT) approaches for BDD in adolescents (Mataix-Cols et al., 2015) involve supporting the young person to challenge their negative cognitions and engage in exposure therapy to feared scenarios, while resisting their usual BDD rituals and/or safety behaviours (known as exposure with response prevention; ERP). Given adolescents with BDD in the current study reported experiencing heightened distress from appearance-based victimisation and lower perceptions of social support from peers, clinicians working with adolescents with BDD should assess the risk of further victimisation when practising ERP in social situations. In cases where ongoing victimisation occurs, in addition to cognitive restructuring and ERP, treatment for BDD should also aim to support the young person, their family, and other stakeholders (e.g., school staff) to reduce victimisation and increase access to social supports. Adolescents with BDD may also benefit from skills training such as coping strategies to deal with distress and assertiveness for dealing with bullying, seeking social support, and establishing new healthy friendships. While further research is needed to understand the relationship between social media use and BDD, results of the current study suggest that clinicians working with adolescent BDD should screen for social media activities that may be a part of symptomatology. For example, therapists can work with the young person and their family to monitor for online behaviours that may be reinforcing their preoccupation and distress, such as online appearance comparisons.

### Limitations

The current study is limited by its reliance on cross-sectional self-report data. Conclusions related to the direction of effects therefore cannot be drawn. For instance, it is unclear whether adolescents are more likely to develop BDD symptoms as a result of experiencing social adversity or whether they have biased perceptions of their social environment. Alternatively, young people with BDD may withdraw socially, or seek out social support less frequently, due to the shame and low confidence associated with their appearance preoccupation (Kuck et al., 2021; Malcolm et al., 2021a; Weingarden et al., 2018). The study is also limited by its relatively small sample comprised predominantly of white, cisgender girls. Moreover, results may be influenced by demand characteristics, whereby participants may have been able to infer the study hypotheses. Hence, future studies utilising multi-informant report (e.g., peers and teachers), distractor tasks (Want, 2014), and longitudinal data in larger clinical samples are needed. Participants in the current study were also self-referred and aware the study included an option for treatment of BDD, meaning they were potentially more motivated and/or educated, limiting the generalisability of findings. Finally, the current study measured social media behaviours, however the motivations behind adolescents’ engagement in these behaviours were not assessed, hence a measure such as the Motivations for Social Media Use scale may be useful in future research (Rodgers et al., 2021).

## Conclusion

The current study is the first to investigate a broad range of peer-related experiences in a clinical sample of adolescents with BDD relative to clinical and non-clinical controls. Findings suggest that distress due to peer appearance victimisation, perceptions of low social support, and online appearance comparisons may be part of the clinical phenomenology of BDD in adolescents. Future research should aim to replicate findings in larger clinical samples, as well as prospective studies to help tease apart whether negative peer experiences and social media activities contribute to the development and/or exacerbation of BDD symptoms in adolescents.
